# Impact of Physical Exercise Alone or in Combination with Cognitive Remediation on Cognitive Functions in People with Schizophrenia: A Qualitative Critical Review

**DOI:** 10.3390/brainsci13020320

**Published:** 2023-02-14

**Authors:** Giacomo Deste, Daniele Corbo, Gabriele Nibbio, Mauro Italia, Dario Dell’Ovo, Irene Calzavara-Pinton, Jacopo Lisoni, Stefano Barlati, Roberto Gasparotti, Antonio Vita

**Affiliations:** 1 Department of Clinical and Experimental Sciences, University of Brescia, 25123 Brescia, Italy; 2 Department of Mental Health and Addiction Services, ASST Spedali Civili of Brescia, 25123 Brescia, Italy; 3 Department of Medical and Surgical Specialties, Radiological Sciences and Public Health, University of Brescia, 25123 Brescia, Italy; 4 Neuroradiology Unit, ASST Spedali Civili of Brescia, 25123 Brescia, Italy

**Keywords:** cognitive remediation, integrated interventions, physical exercise, psychiatric rehabilitation, psychosocial interventions, schizophrenia, cognitive functioning, recovery

## Abstract

Physical exercise and cognitive remediation represent the psychosocial interventions with the largest basis of evidence attesting their effectiveness in improving cognitive performance in people living with schizophrenia according to recent international guidance. The aims of this review are to provide an overview of the literature on physical exercise as a treatment for cognitive impairment in schizophrenia and of the studies that have combined physical exercise and cognitive remediation as an integrated rehabilitation intervention. Nine meta-analyses and systematic reviews on physical exercise alone and seven studies on interventions combining physical exercise and cognitive remediation are discussed. The efficacy of physical exercise in improving cognitive performance in people living with schizophrenia is well documented, but more research focused on identifying moderators of participants response and optimal modalities of delivery is required. Studies investigating the effectiveness of integrated interventions report that combining physical exercise and cognitive remediation provides superior benefits and quicker improvements compared to cognitive remediation alone, but most studies included small samples and did not explore long-term effects. While physical exercise and its combination with cognitive remediation appear to represent effective treatments for cognitive impairment in people living with schizophrenia, more evidence is currently needed to better understand how to implement these treatments in psychiatric rehabilitation practice.

## 1. Introduction

Schizophrenia (SCZ) represents a severe mental disorder which is often associated with poor functional outcomes [1,2,3]. Cognitive impairment is one of the core features of the disorder and has been considered of great relevance since its earliest conceptualizations [4,5]. In fact, deficits in both neurocognitive performance [6] and social cognition abilities [7] can be frequently observed in people living with SCZ. Cognitive deficits are one of the core determinants of functional impairment, showing an important negative impact on both functional capacity and real-world functioning [8,9,10], and they also represent one of the main limiting factors for the process of recovery in the context of psychiatric rehabilitation [11,12,13,14].

Pharmacological treatment is effective in reducing the core symptoms of SCZ [15,16], but its impact on cognition appears to be limited, as available medications provide minimal effects on cognitive functioning [17,18]. This has led to a considerable scientific and clinical interest regarding the potential usefulness of non-pharmacological interventions [3,19].

According to the most recent European Psychiatric Association guidance, the psychosocial interventions that have the most solid body of evidence confirming their effectiveness in improving cognitive performance in people living with SCZ are physical exercise (PE) and cognitive remediation (CR) [18].

PE can be considered an evidence-based intervention, as it provides consistent benefits for people living with mental disorders, and in people living with SCZ, physical activity is currently recommended as an adjunctive treatment in rehabilitation programs to improve symptoms severity and also overall quality of life [20,21]. While PE could also provide substantial benefits to cognitive performance, its moderators of effectiveness and the optimal modalities to deliver it in clinical practice to specifically target cognitive impairment in SCZ currently require better understanding.

CR is a behavioral training-based intervention that specifically targets cognitive processes and that has proven to be effective also in improving functional outcomes [22,23,24,25], and which appears to be more effective in the real-world context when implemented into structured rehabilitation programs or alongside other evidence-based treatments [23,26,27,28].

As both these interventions appear to be characterized by good practical feasibility and relatively low resource cost for implementation in rehabilitation practice, better understanding the extent of their effectiveness and their potential as a combined treatment program could represent a topic of considerable clinical interest.

The aims of the present narrative review are to provide a comprehensive overview of the literature attesting the effectiveness of PE as a treatment for cognitive impairment in people living with SCZ and of the studies that have combined PE and CR as an integrated rehabilitation intervention.

## 2. Methods

To ensure a comprehensive assessment of the relevant literature, works included in the present narrative review were drawn from a search conducted in open databases (PubMed, Scopus, and Google Scholar) with combinations of the following research terms: [physical AND (exercise OR activity)], [cognitive AND (remediation OR rehabilitation OR training)] and (schizophrenia OR psychosis). Given the substantial number on studies of the effects on PE on cognition in people living with SCZ, only systematic reviews and meta-analyses were considered for the first part of the present work, while individual studies were considered for interventions combining PE and cognitive remediation.

Results of this search were also checked against the results of a recent European Psychiatric Association guidance document on treatment of cognitive impairment [18] and from the systematic search conducted for a recent and comprehensive systematic review and meta-analysis on CR trials [29].

## 3. Results

### 3.1. Effectiveness of Physical Exercise on Cognition in Schizophrenia

In recent years, an ever-growing body of literature exploring the relationship between PE and cognition in people living with SCZ has been published, posing an interesting background for future research (Table 1).

Exploring the literature in chronological order, we found that in a 2012 systematic review including 10 randomized clinical trials (RCT) (for a total of 322 SCZ participants), it is stated that aerobic and strength exercises have been proven to be effective in improving short-term memory, a result that was correlated to an increase in the volume of the hippocampus [30].

Then, in 2015, Firth et al. analyzed 17 trials (for a total of 659 non-affective psychotic patients), finding a positive effect of exercise on neurocognition and, in particular, in one study it was observed that exercise was associated to improvements in short-term verbal memory of 34% (*p* < 0.05) and to increase in brain volume of 12% (significantly more than that in one of the control groups) [32].

The next year, a Dutch review with broader inclusion criteria selected 29 studies (for a total of 1109 SCZ patients) and found that exercise in general had no demonstrated effect on cognition, with the exception of yoga which was seen to improve the cognitive subdomain of long-term memory (n = 184: Hedges’ g = 0.32, *p* < 0.05), and with an additional trend towards significance for the subdomains of attention and executive functioning (n = 184: Hedges’ g = 0.38, *p* = 0.08) [31].

In 2017, the group of Firth et al. published a review including 16 trials (for a total of 423 SCZ patients) of which 7 focused on neuroimaging: preliminary evidence indicated that cognitive improvements provided by PE for people living with SCZ are related to improvements in brain structure and connectivity, and some of these studies found a positive correlation between brain-derived neurotrophic factor (BDNF) and cognitive enhancements, indicating that neurogenesis could be the mechanism underlying the cognitive benefits of exercise in SCZ (current evidence, however, is too limited to provide definitive conclusions) [33].

In the same year, Firth and collaborators also conducted a meta-analysis presenting evidence that PE significantly improves cognition and that interventions using “higher dosages” of PE are related to greater improvements in global cognition. Moreover, subgroup analyses suggested that interventions including direct supervision from a physical activity instructor resulted in larger cognitive improvement. Moreover, domain-specific analyses highlighted that attention/vigilance, working memory, and social cognition had substantial improvements: all these domains are among those most commonly impaired in people living with SCZ and represent well-known predictors of real-world outcomes. Finally, the authors report that aerobic exercise (AE) may be more effective than yoga for cognitive outcomes in SCZ, unlike what was observed in previous research [34].

The next year, in 2018, a systematic review by Li et al. explored the efficacy of mindful (i.e., yoga, different forms of Tai Chi, and Qigong) versus non-mindful exercise in subjects with SCZ: the former came out as being significantly more effective than the latter in the accuracy index for working memory, but the RCT (n = 194) coming to the conclusion is described by Li et al. as being low-quality (MD = 0.39), whereas in the domains of “attention” and “social functioning”, no clear difference was found (MD = −0.48) [35].

In the same year, a Dutch systematic review comparing the effects of AE in people living with a SCZ spectrum disorder (168 subjects) and healthy participants (for a total of 201 subjects) within a similar age range was published. Most studies included in this work effects on hippocampal volume, reporting positive outcomes: PE could help in preventing the reduction of hippocampal volume over time in people living with SCZ; in healthy subjects, changes in connectivity of the dorsolateral prefrontal cortex related to better cognitive performance were reported. In the comparisons between participants with SCZ spectrum disorders and healthy subjects, similar exercise-mediated effects with a dose–response relationship on hippocampal volume as well as on white matter tracts were observed. However, these effects appear to be smaller in people living with SCZ spectrum disorders. Finally, this systematic review underlines the need for further studies to investigate additional neural correlates beside the hippocampus [36].

In 2021, then, a meta-analysis by Dauwan et al. (including studies on neurological conditions such as Alzheimer’s disease, Parkinson’s disease, Huntington’s disease, and multiple sclerosis, as well as mental disorders such as SCZ and major depressive disorder) showed that exercise provided greater gains than treatment as usual (TAU) in several cognitive domains, including attention and working memory (*p* < 0.009), executive functioning (*p* = 0.013), memory (*p* = 0.038), and psychomotor speed (*p* = 0.003), and these results were proven not to be age-dependent [37].

In the same year, a Spanish research team published a meta-analysis of 59 RCTs analyzing interventions on diet and physical activity in people living with non-affective psychosis, including also participants with first-episode psychosis (FEP). Significant improvements were observed for many cognitive domains: two studies evaluated short-term memory performance and attention through the WAIS’ forward and backward digit span tests, with positive post-treatment effects for both measures (g = 0.309, *p* = 0.014 for the forward test and g = 0.621, *p* < 0.001 for the backward test), although neither had an effect that persisted at follow-up (g = −0.132, *p* = 0.528 and g = −0.201, *p* = 0.291, respectively). Furthermore, different studies explored other outcomes such as short- and long-term memory measured through the Rey Auditory Verbal Learning Test (RAVLT) (significant only for the short-term memory component); short-term visual memory measured through the Corsi direct block span (not significant); attention, spatial memory, and working memory measured via the Penn computerized neurocognitive battery (several domains showed a significant improvement for both yoga and other types of exercise, some persisting at follow-up as well); executive functions measured through the Wisconsin Card Sorting Test (WCST) (showing a significant improvement); attention, memory, social cognition, and other domains measured through the MATRICS Consensus Cognitive Battery (showing improvement in the scores correlated to improvement in physical performances); motor and sequencing skills measured through the Neurological Evaluation Scale (showing improvements after exercise interventions that were not maintained at follow-up); and speed of processing, working memory, verbal learning, and visual learning measured through a variety of tests (only working memory was significantly improved after the intervention). Only two studies found no post-intervention effect on cognition [38].

### 3.2. Combination of Physical Exercise and Cognitive Remediation for Treating Cognition in Schizophrenia

Both CR and PE have been reported to be effective on improving cognition in SCZ: several works in the recent literature compare the efficacy of the two interventions and explore whether they have a synergistic effect. In the next paragraph, we present the relevant literature in a chronological order. A summary of the studies can be found in Table 2.

In 2013, Tan and King published their RCT on the effects of CR on functional outcomes in people living with SCZ. A total of 70 included participants were allocated to 2 groups: 34 subjects were randomized to the PE group, whereas 36 were randomized to the CR group. Neurocognitive and functional outcomes were measured at baseline, after 3 months (end of treatment), after 9 months, and after 1 year and 3 months from baseline. Over time, the CR group kept a greater improvement on all measures of neurocognition when compared to the PE group (*p* < 0.05 for all tests: Comprehensive Trail Making Test, RAVLT, Wechsler Adult Intelligence Scale’s Digit Span Forward and Backward tests) [39].

In 2014, Oertel-Knöchel et al. published a study focusing on the effects on cognition of a combination of PE and cognitive training (CT) in SCZ (SCZ, n = 29) and major depressive disorder (MDD, n = 22). Patients were randomly allocated in the following groups: CT + PE (n = 16, of which 8 diagnosed with SCZ); CT + relaxation exercises (n = 17, of which 11 diagnosed with SCZ); and “waiting control group” to check for potential biases (n = 18, of which 10 diagnosed with SCZ). Amelioration of the cognitive performances was observed in both treatment groups, although the improvement was greater in the group undergoing CT + AE. Specifically, the results showed a significant post-intervention increase in performances in the cognitive subdomains of speed of processing (*p* < 0.001), working memory (*p* = 0.01), and visual learning (*p* = 0.004). Additionally, the results showed that the improvement in cognition was greater in patients living with SCZ compared to people suffering from MDD, suggesting that the combined intervention is even more effective in SCZ (*p* < 0.001) [40].

Malchow et al. conducted a single-center trial, taking place between 2010 and 2013 and evaluating the effects of bicycle ergometer training and add-on computer-assisted remediation (CACR) training, compared to a control group playing table tennis + CACR. The study included 22 subjects with SCZ and 22 healthy controls matched for age and sex; all the subjects underwent 3 months of endurance training (30 min, three times/week) and CACR training (30 min, two times/week) was added from week 6. An additional group including 21 subjects living with SCZ played table soccer and additionally received the same CACR training. The performance in the Verbal Learning Memory Test (VLMT) and WCST improved significantly in subjects with SCZ belonging to the endurance training + CACR group. The positive effects were specific to the endurance training + CACR group and were not observed in patients playing table soccer + CACR [41].

A pilot study conducted in 2016 by Neuchterlein et al. was designed to test the efficacy of enhancing CT with AE in patients with a recent onset of psychotic illness. A total of 18 patients were enrolled into two different groups: 7 subjects were allocated to the Cognitive Training & Exercise (CT&E) group and 9 to CT alone group for a 10-week period. All patients received treatment with a second-generation antipsychotic medication and regular psychiatric visits in addition to the study interventions. The effect size for the improvement in the MCCB score was larger for CT&E patients when compared to CT patients: Cohen’s f is 0.48 and the single cognitive domains with the largest differential gains for CT&E (when compared to CT alone) were social cognition (f = 0.65), working memory (f = 0.50), speed of processing (f = 0.38), and attention/vigilance (f = 0.33) [42].

In 2020, Choi et al. randomly allocated 85 outpatients diagnosed with SCZ into 2 groups of 18 hours of either PE (29 subjects), CT focused on processing speed and working memory (27 subjects), or a time-matched combination of the two (29 subjects). Working memory was seen to be improved post-intervention in all groups, with greater gains in the PE only group, and to a lesser extent in the PE + CT group, when compared to the CT only group (*p* < 0.001). After 3 months from the end of the treatment instead, the only group still showing significant improvements in working memory was the CT + PE group (*p* < 0.001). Moreover, concerning processing speed, after the intervention, all groups showed better scores, but the only group showing a significant improvement compared to the others was the PE only group (*p* < 0.001), whereas at follow-up, the only group showing a persistent improvement compared to the other groups was the PE + CT group (*p* < 0.001) [43].

A very recent work by Neuchterlein et al. on the same topic explored the effect of a PE + CT program lasting 6 months compared to CT in 47 outpatients with FEP. The combination of AE and CT led to faster cognitive improvement and to larger gains in psychosocial functioning compared to CT. Both groups improved in a six-month period of CT treatment, but AE appeared to provide an additional boost to CT in the first three months. BDNF increase appeared to be associated with the dimension of cognitive improvement, but this association was observed only at a statistical trend-level. The authors reflect upon the possibility that people with FEP could be more motivated to participate in PE and present fewer medical comorbidities, limiting the access to PE than people living with more advanced stages of SCZ; the randomized treatments were delivered in the context of a psychiatric rehabilitation program, and this could facilitate larger effects on cognitive performance and its transfer to real-world functioning. The view that the quantity of completed exercise represented an essential factor in the cognitive improvement observed in the PE + CT group is backed also by correlations observed between improvements in cognitive performance and proportion of completed exercise (r = 0.56) and the number of homework sessions (r = 0.61). The transfer of improvement in cognitive performance to real-world functioning appeared to be related to the amount of completed exercise (r = 0.51), but it also appeared to be associated with the amount of participation in CT sessions as well as in bridging groups. These findings support the hypothesis that exercise represents the core factor behind cognitive improvement, while participation in other components of the intervention is responsible for improving generalization to real-world functioning [44].

Very recently, Dai et al. published an RCT comparing 3 groups of individuals living with SCZ: 31 subjects in the treatment as usual group, 26 subjects in the computerized CR therapy + aerobic exercise (CAE) group, and 25 subjects in the AE group. From the results, it is reported that the CAE group had a significantly better performance in terms of change of processing speed and cognitive flexibility scores when compared to the control and the AE groups after treatment (*p* < 0.05) [45].

## 4. Discussion

Considering the emerging evidence, there is a clear rationale to propose PE intervention to people living with SCZ.

PE interventions delivered in trials recruiting people living with SCZ and considering cognitive performance among the study outcomes included a wide variety of different types of PE. Among these, the most represented were AE, anaerobic exercise (also defined as strength exercise), mind-body exercise (also defined as mindful exercise, encompassing exercise such as yoga, Tai Chi, and Qigong), and dance movement therapy. AE included both light exercise requiring low consumption of energy, such as walking or light intensity exercise in standing or sitting position, and moderate or vigorous exercise requiring high energy consumption for several minutes, such as high intensity cycling, dancing, jogging, and running; some studies combined both AE and anaerobic exercise. Frequency of administration varied considerably between studies, ranging from 60 min once per week to 45–60 min three times per week for AE and anaerobic exercise; mind-body exercise interventions were implemented with higher intensity, up to 90 min seven times per week. The duration of the program also varied considerably, ranging from 4 to 56 weeks.

According to the results of several meta-analysis and systematic reviews, PE-based interventions could not only provide substantial benefits for metabolic, endocrinological, and health-related reasons, but also provide reliable improvement in cognitive performance.

In fact, several cognitive domains that are frequently impaired in people living with SCZ, including attention, short- and long-term memory, executive functions, working memory, and social cognition abilities, showed consistent gains in systematic reviews and meta-analytic evidence investigating the effects of PE.

In this regard, PE can be fully considered an evidence-based intervention for treatment of cognitive impairment in SCZ [18,21].

However, despite the wealth of the literature attesting its efficacy, moderators of participants response and optimal modalities of delivery of psychical exercise-based interventions still require to be further explored. For instance, in one of the most recent and well-conducted meta-analyses [34], no significant moderator of response emerged besides a trend–level relationship between greater amounts of exercise in minutes of activity per week of treatment and greater cognitive gains. Another recent meta-analysis [38] reported similar results, as it found a small superior effect of moderate-to-vigorous AE compared to light AE or mind-body exercise.

While these observations further confirm the clinical effectiveness of PE, as they show a dose–response effect, they do not provide useful insight to improve its usefulness in a clinical context and to better implement it in rehabilitation settings. In this regard, future research should focus on better identifying ideal candidates for this treatment, the optimal duration and intensity of programs, as well as barriers and facilitators for its implementation in rehabilitation settings [46].

As CR represents the psychosocial intervention with the highest level of recommendation in recent international guidance [18], and it appears to provide greater benefits on both cognitive and functional outcomes when combined with other interventions [23,28], pairing it with psychical exercise as a combined treatment appears to be a potentially effective strategy.

To date, to the best of our knowledge, no systematic assessment and meta-analysis focusing on interventions combining CR and PE has been performed. However, several individual studies, independently conducted in different research and clinical centers, have assessed the efficacy of this combination.

Most of these studies report a superior effect on cognitive outcomes of combined treatment compared to CR alone or to non-specific control conditions; one study in particular reported that adding PE appears to provide a ‘boosting effect’ on cognitive performance, leading to faster gains [44].

While these results are very interesting, it should also be pointed out that all of these studies included small samples of participants, so their results must be considered preliminary and do not offer a definite proof of superior efficacy or effectiveness of this combined treatment.

Instead, these promising results should represent the conceptual basis for additional confirmatory studies, including larger samples and conducted with a multicentric approach.

Moreover, long-term effectiveness of PE and combined interventions requires further study: durability of positive effects is essential if cognitive gains are to be translated into real-world outcomes [26,47], and while the results of several studies including follow-up observations suggest that CR does indeed provide lasting benefits [48,49,50,51], the long-term effect of combined approaches remains to be better explored, and their long-term superiority to CR alone remains to be demonstrated.

Finally, moderators of response of this combined treatment also remain to be further explored: possible moderators include both participant-related characteristics, such as duration of illness, duration of untreated psychosis, age of onset of the disorder, baseline cognitive functioning, and baseline symptoms severity, and treatment-related features, such as duration and intensity of both CR and PE, the presence of an active and trained therapist, and an individual or group delivery of treatment. Several of these factors appear to affect participants’ response to CR interventions [22,23], but their impact on a combined treatment requires further research.

The present review has some notable points of strength. It presents a solid element of novelty, as it provides both a comprehensive update on systematic works that investigate the effectiveness of PE-based interventions on cognition in people living with SCZ and is, to the best of our knowledge, the first review to provide a collection of all studies integrating CR and PE interventions. As cognitive performance represents one of the stronger predictors of real-world rehabilitation outcomes and is also one of the main treatment targets of importance in the patient’s perspective [52,53], providing further insight on effective ways to improve it represents an issue of clear scientific and clinical interest.

However, some limitations have to be acknowledged. As a narrative review, the studies summarized and discussed were not identified through a systematic search strategy, which was beyond the scope of the present work and may represent an interesting perspective for future research. Moreover, no quantitative analysis was performed for combined treatments; however, considering the wide heterogeneity of designs and control conditions of reported studies, a pooled effect estimate might not be sufficiently accurate, and certainty of the provided evidence may not be sufficient.

Despite these limitations, the reviews and the studies offer valuable insight on a topic of considerable scientific interest, highlighting that steps are required in clinical research to further improve cognitive and rehabilitation outcomes of people living with SCZ.

## 5. Conclusions

Several systematic reviews and meta-analysis attest to the efficacy of PE in improving cognitive performance in people living with SCZ, but moderators of participants response and optimal modalities of delivery remain to be identified.

Integrated interventions combining CR and PE appear to provide superior benefits and quicker improvements compared to cognitive radiation alone, but larger studies are required to confirm these findings and longitudinal observations are necessary to attest the durability of positive effects.

Future research should also focus on better understanding the barriers and facilitators for the implementation in rehabilitation practice of both PE and combined interventions.

## Figures and Tables

**Table 1:** Studies on the effect of physical exercise on cognition in schizophrenia.

Year of Publication and Authors	Type of Study	Number of Included Studies (Total Number of Participants)	Summary
Vancampfort et al., 2012 [30]	Systematic Review	10 (322)	In one study, STM (correlated to an increased hippocampal volume) is improved by both AE and SE.
Dauwan et al., 2016 [31]	Meta-analysis	17 (659)	In one study, exercise improved verbal STM by 34% (*p* < 0.05) and brain volume was increased by 12%.
Firth et al., 2015 [32]	Meta-analysis	29 (1109)	Only yoga seems to influence the subdomain of LTM (*p* < 0.05), and to have a trend towards significance for AT and EF (*p* = 0.08).
Firth et al., 2017a [33]	Systematic Review	16 (423)	Improvements in brain structure and connectivity are associated to exercise’s beneficial effects on cognition in SCZ. Some studies also found a positive correlation between BDNF and cognitive enhancements.
Firth et al., 2017b [34]	Meta-analysis	10 (385)	PE significantly improves cognition and “higher dosages” of PE and supervised PE are associated with larger improvements in global cognition. Moreover, PE is particularly beneficial for SC, WM, and AT. Finally, AE seems more effective than yoga.
Li et al., 2018 [35]	Systematic Review	7 (679)	In one RCT (n = 194, low quality), mindful exercise is significantly more effective than other forms of exercise in WM subtests (MD 0.39).
Van der Stouwe et al., 2018 [36]	Systematic Review	29 (4448)	The protective effects of exercise on the reduction of hippocampal volume and white matter tracts were similar for both SSD and HC and they showed a dose–response relationship, but the effects in people with SSD appear to be of reduced entity.
Dauwan et al., 2021 [37]	Meta-analysis	122 (7231)	PE was superior to TAU in improving AT and WM (*p* < 0.009), EF (*p* = 0.013), memory (*p* = 0.038), and PS (*p* = 0.003). Results were proven not to be age-dependent.
Fernández-Abascal et al., 2021 [38]	Meta-analysis	59 (4202)	Post-intervention benefit was found for many cognitive domains: STM and AT measured through the WAIS’ forward and backward DSTs showed a significant effect at the end of the intervention (*p* = 0.014 for the forward test and *p* < 0.001 for the backward test), although the effects did not persist at follow-up (*p* = 0.528 and *p* = 0.291, respectively). Furthermore, different studies explored other outcomes through different measures: through the RAVLT test (significant only for the STM); through the Corsi direct block span for visual STM (not significant); through the Penn computerized neurocognitive battery for attention, SM, and WM (several domains showed a significant improvement for both yoga and other types of exercise, some persisting at follow-up as well); through the WCST for EF (significant improvement); through the MCCB for AT, memory, and SC (improvements seen in the scores correlated to improvement in physical performances); through the NES test for motor and sequencing skills (showing improvement but not maintained at follow-up); and other miscellaneous tests for SP, WM, VeL, and ViL (only WM was significantly improved). Only two studies found no post-intervention effects on cognition.

AE (aerobic exercise), AT (attention), DST (digit span test), EF (executive functioning), HC (healthy controls), LTM (long-term memory), MCCB (MATRICS Consensus Cognitive Battery), NES (Neurological Evaluation Scale), PE (physical exercise), PS (psychomotor speed), RCT (randomized controlled trial), RAVLT (Rey Auditory Verbal Learning Test), SC (social cognition), SCZ (schizophrenia), SE (strength exercise), SM (spatial memory), SP (speed of processing), SSD (schizophrenia spectrum disorders), STM (short-term memory), TAU (treatment as usual), VeL (verbal learning), ViL (visual learning), WAIS (Weschler Adult Intelligence Scale), WCST (Wisconsin Card Sorting Test), WM (working memory).

**Table 2:** Studies on the effect of physical exercise in combination with cognitive remediation on cognition in schizophrenia.

Year of Publication and Authors	Type of Study	Groups	Total Number of Participants (Diagnosis)	Summary
Tan and King, 2013 [39]	RCT	PE and CR	70 (SCZ)	The CR group kept a greater improvement in all of neurocognitive tests when compared to the PE group (*p* < 0.05 for all tests).
Oertel-Knöchel et al., 2014 [40]	RCT	CR+AE, CR+RT, WW	51 (29 SCZ + 22 MDD)	Cognitive performances were improved in both treatment groups (CT+AE group better than CT+RT), particularly in the cognitive subdomains of SP (*p* < 0.001), WM (*p* = 0.01), and ViL (*p* = 0.004). Additionally, the results show that the improvement is greater in SCZ compared to MDD, suggesting that the combined intervention is even more effective in SCZ (*p* < 0.001).
Malchow et al., 2015 [41]	Clinical Trial	PE, PE + CACR, TT + CACR	65 (43 SCZ + 22 HC)	The performance in VLMT and WCST significantly improved in the PE + CACR group. The positive effects were not seen in the TT + CACR group.
Nuechterlein et al., 2016 [42]	Pilot Study	CT&E, CT	18 (recent onset SSD)	The ES for the improvement in the MCCB score was larger for CT&E patients when compared to CT patients: the largest differential gains that were seen for CT&E were SC (f = 0.65), WM (f = 0.50), SP (f = 0.38), and attention (f = 0.33).
Choi et al., 2019 [43]	RCT	PE, CT, PE + CT	85 (SCZ)	WM was improved in all intervention groups (vs. the CT only group) and mostly in the PE only group (*p* < 0.001). At follow-up instead, the only group still showing significant improvements in WM was the CT + PE group (*p* < 0.001). Moreover, concerning PS, the only group showing a more significant improvement (vs. other groups) was the PE only group (*p* < 0.001), whereas at follow-up, only the PE + CT group (*p* < 0.001).
Nuechterlein et al., 2022 [44]	RCT	CT&E, CT	47 (FEP)	PE + CT (compared to CT alone) provided faster gains in cognition and greater improvements in psychosocial functioning. Strong correlations were observed between cognitive gains at six months and proportion of PE completed (r = 0.56) and the number of homework PE sessions (r = 0.61). The transfer of cognitive improvement to real-world functioning appears to be related to the quantity of completed PE (r = 0.51), and to participation in both CT sessions (r = 0.46) and bridging groups (r = 0.56).
Dai et al., 2022 [45]	RCT	CAE, AE, TAU	82 (SCZ)	The CAE group had a significantly better performance in PS and cognitive flexibility when compared to the TAU and the AE groups (*p* < 0.05).

AE (aerobic exercise), CACR (computer-assisted cognitive remediation), CAE (cognitive remediation + aerobic exercise), CR (cognitive remediation), CT (cognitive training), CT&E (Cognitive Training & Exercise), ES (effect size), FEP (first episode psychosis), HC (healthy controls), MCCB (MATRICS Consensus Cognitive Battery), PE (physical exercise), RCT (randomized controlled trial), RT (relaxation training), SC (social cognition), SCZ (schizophrenia), SP (speed of processing), SSD (schizophrenia spectrum disorders), TAU (treatment as usual), TT (table tennis), ViL (visual learning), VLMT (Verbal Learning Memory Test), WCST (Wisconsin Card Sorting Test), WM (working memory), WW (wait and watch).

## Data Availability

Not applicable.

## References

[1] Galderisi S., Rossi A., Rocca P., Bertolino A., Mucci A., Bucci P., Rucci P., Gibertoni D., Aguglia E., Amore M. (2014). The influence of illness-related variables, personal resources and context-related factors on real-life functioning of people with schizophrenia. World Psychiatry.

[2] Harvey P.D., Strassnig M. (2012). Predicting the severity of everyday functional disability in people with schizophrenia: Cognitive deficits, functional capacity, symptoms, and health status. World Psychiatry.

[3] Maj M., Van Os J., De Hert M., Gaebel W., Galderisi S., Green M.F., Guloksuz S., Harvey P.D., Jones P.B., Malaspina D. (2021). The clinical characterization of the patient with primary psychosis aimed at personalization of management. World Psychiatry.

[4] Bleuler E. (1908). Die Prognose Der Dementia Praecox (Schizophreniegruppe). Allg. Z. Psychiat.

[5] Kraepelin E., Barclay R. (1919). Dementia Praecox. The Clinical Roots of the Schizophrenia Concept: Translations of Seminal European Contributions on Schizophrenia.

[6] Green M.F., Horan W.P., Lee J. (2019). Nonsocial and social cognition in schizophrenia: Current evidence and future directions. World Psychiatry.

[7] Pinkham A.E., Harvey P.D., Penn D.L. (2018). Social Cognition Psychometric Evaluation: Results of the Final Validation Study. Schizophr. Bull..

[8] Deste G., Vita A., Nibbio G., Penn D.L., Pinkham A.E., Harvey P.D. (2020). Autistic Symptoms and Social Cognition Predict Real-World Outcomes in Patients With Schizophrenia. Front. Psychiatry.

[9] Fett A.-K.J., Viechtbauer W., Dominguez M.-D., Penn D.L., van Os J., Krabbendam L. (2010). The relationship between neurocognition and social cognition with functional outcomes in schizophrenia: A meta-analysis. Neurosci. Biobehav. Rev..

[10] Galderisi S., Rucci P., Kirkpatrick B., Mucci A., Gibertoni D., Rocca P., Rossi A., Bertolino A., Strauss G.P., Aguglia E. (2018). Interplay Among Psychopathologic Variables, Personal Resources, Context-Related Factors, and Real-life Functioning in Individuals With Schizophrenia: A Network Analysis. JAMA Psychiatry.

[11] Giordano G.M., Galderisi S., Pezzella P., Perrottelli A., Bucci P., Carpiniello B., Vita A., Mencacci C. (2022). Determinants of Clinical Recovery in Schizophrenia. Recovery and Major Mental Disorders.

[12] Spaulding W.D., Sullivan M.E. (2016). Treatment of Cognition in the Schizophrenia Spectrum: The Context of Psychiatric Rehabilitation. Schizophr. Bull..

[13] Vita A., Barlati S. (2018). Recovery from schizophrenia. Curr. Opin. Psychiatry.

[14] Wykes T., Dunn G. (1992). Cognitive deficit and the prediction of rehabilitation success in a chronic psychiatric group. Psychol. Med..

[15] Galderisi S., Mucci A., Dollfus S., Nordentoft M., Falkai P., Kaiser S., Giordano G.M., Vandevelde A., Nielsen M., Glenthøj L.B. (2021). EPA guidance on assessment of negative symptoms in schizophrenia. Eur. Psychiatry.

[16] Leucht S., Leucht C., Huhn M., Chaimani A., Mavridis D., Helfer B., Samara M., Rabaioli M., Bächer S., Cipriani A. (2017). Sixty Years of Placebo-Controlled Antipsychotic Drug Trials in Acute Schizophrenia: Systematic Review, Bayesian Meta-Analysis, and Meta-Regression of Efficacy Predictors. Am. J. Psychiatry.

[17] Veselinović T., Neuner I. (2022). Progress and Pitfalls in Developing Agents to Treat Neurocognitive Deficits Associated with Schizophrenia. CNS Drugs.

[18] Vita A., Gaebel W., Mucci A., Sachs G., Barlati S., Giordano G.M., Nibbio G., Nordentoft M., Wykes T., Galderisi S. (2022). European Psychiatric Association guidance on treatment of cognitive impairment in schizophrenia. Eur. Psychiatry.

[19] Solmi M., Croatto G., Piva G., Rosson S., Fusar-Poli P., Rubio J.M., Carvalho A.F., Vieta E., Arango C., DeTore N.R. (2022). Efficacy and acceptability of psychosocial interventions in schizophrenia: Systematic overview and quality appraisal of the meta-analytic evidence. Mol. Psychiatry.

[20] Ashdown-Franks G., Firth J., Carney R., Carvalho A.F., Hallgren M., Koyanagi A., Rosenbaum S., Schuch F.B., Smith L., Solmi M. (2020). Exercise as Medicine for Mental and Substance Use Disorders: A Meta-review of the Benefits for Neuropsychiatric and Cognitive Outcomes. Sports Med..

[21] Stubbs B., Vancampfort D., Hallgren M., Firth J., Veronese N., Solmi M., Brand S., Cordes J., Malchow B., Gerber M. (2018). EPA guidance on physical activity as a treatment for severe mental illness: A meta-review of the evidence and Position Statement from the European Psychiatric Association (EPA), supported by the International Organization of Physical Therapists in Mental Health (IOPTMH). Eur. Psychiatry.

[22] Lejeune J.A., Northrop A., Kurtz M.M. (2021). A Meta-analysis of Cognitive Remediation for Schizophrenia: Efficacy and the Role of Participant and Treatment Factors. Schizophr. Bull..

[23] Vita A., Barlati S., Ceraso A., Nibbio G., Ariu C., Deste G., Wykes T. (2021). Effectiveness, Core Elements, and Moderators of Response of Cognitive Remediation for Schizophrenia: A Systematic Review and Meta-Analysis of Randomized Clinical Trials. JAMA Psychiatry.

[24] Wykes T., Huddy V., Cellard C., McGurk S.R., Czobor P. (2011). A Meta-Analysis of Cognitive Remediation for Schizophrenia: Methodology and Effect Sizes. Am. J. Psychiatry.

[25] Yeo H., Yoon S., Lee J., Kurtz M.M., Choi K. (2021). A meta-analysis of the effects of social-cognitive training in schizophrenia: The role of treatment characteristics and study quality. Br. J. Clin. Psychol..

[26] Bowie C.R., Bell M.D., Fiszdon J.M., Johannesen J.K., Lindenmayer J.-P., McGurk S.R., Medalia A.A., Penadés R., Saperstein A.M., Twamley E.W. (2020). Cognitive remediation for schizophrenia: An expert working group white paper on core techniques. Schizophr. Res..

[27] Montemagni C., Del Favero E., Riccardi C., Canta L., Toye M., Zanalda E., Rocca P. (2021). Effects of Cognitive Remediation on Cognition, Metacognition, and Social Cognition in Patients With Schizophrenia. Front. Psychiatry.

[28] Nibbio G., Barlati S., Cacciani P., Corsini P., Mosca A., Ceraso A., Deste G., Vita A. (2020). Evidence-Based Integrated Intervention in Patients with Schizophrenia: A Pilot Study of Feasibility and Effectiveness in a Real-World Rehabilitation Setting. Int. J. Environ. Res. Public Health.

[29] Vita A., Barlati S., Ceraso A., Deste G., Nibbio G., Wykes T. (2022). Acceptability of cognitive remediation for schizophrenia: A systematic review and meta-analysis of randomized controlled trials. Psychol. Med..

[30] Vancampfort D., Probst M., Skjaerven L.H., Catalán-Matamoros D., Gyllensten A.L., Gómez-Conesa A., Ijntema R., De Hert M. (2012). Systematic Review of the Benefits of Physical Therapy Within a Multidisciplinary Care Approach for People With Schizophrenia. Phys. Ther..

[31] Dauwan M., Begemann M.J.H., Heringa S.M., Sommer I. (2015). Exercise Improves Clinical Symptoms, Quality of Life, Global Functioning, and Depression in Schizophrenia: A Systematic Review and Meta-analysis. Schizophr. Bull..

[32] Firth J., Cotter J., Elliott R., French P., Yung A.R. (2015). A systematic review and meta-analysis of exercise interventions in schizophrenia patients. Psychol. Med..

[33] Firth J., Cotter J., Carney R., Yung A.R. (2017). The pro-cognitive mechanisms of physical exercise in people with schizophrenia. Br. J. Pharmacol..

[34] Firth J., Stubbs B., Rosenbaum S., Vancampfort D., Malchow B., Schuch F., Elliott R., Nuechterlein K.H., Yung A.R. (2016). Aerobic Exercise Improves Cognitive Functioning in People With Schizophrenia: A Systematic Review and Meta-Analysis. Schizophr. Bull..

[35] Li J., Shen J., Wu G., Tan Y., Sun Y., Keller E., Jiang Y., Wu J. (2018). Mindful exercise versus non-mindful exercise for schizophrenia: A systematic review and meta-analysis of randomized controlled trials. Complement. Ther. Clin. Pract..

[36] van der Stouwe E., van Busschbach J., de Vries B., Cahn W., Aleman A., Pijnenborg G. (2018). Neural correlates of exercise training in individuals with schizophrenia and in healthy individuals: A systematic review. NeuroImage Clin..

[37] Dauwan M., Begemann M.J.H., Slot M.I.E., Lee E.H.M., Scheltens P., Sommer I.E.C. (2019). Physical exercise improves quality of life, depressive symptoms, and cognition across chronic brain disorders: A transdiagnostic systematic review and meta-analysis of randomized controlled trials. J. Neurol..

[38] Fernández-Abascal B., Suárez-Pinilla P., Cobo-Corrales C., Crespo-Facorro B., Suárez-Pinilla M. (2021). In-and outpatient lifestyle interventions on diet and exercise and their effect on physical and psychological health: A systematic review and meta-analysis of randomised controlled trials in patients with schizophrenia spectrum disorders and first episode of psychosis. Neurosci. Biobehav. Rev..

[39] Tan B.-L., King R. (2013). The effects of cognitive remediation on functional outcomes among people with schizophrenia: A randomised controlled study. Aust. N. Z. J. Psychiatry.

[40] Oertel-Knöchel V., Mehler P., Thiel C., Steinbrecher K., Malchow B., Tesky V., Ademmer K., Prvulovic D., Banzer W., Zopf Y. (2014). Effects of aerobic exercise on cognitive performance and individual psychopathology in depressive and schizophrenia patients. Eur. Arch. Psychiatry Clin. Neurosci..

[41] Malchow B., Keller K., Hasan A., Dörfler S., Schneider-Axmann T., Hillmer-Vogel U., Honer W.G., Schulze T.G., Niklas A., Wobrock T. (2015). Effects of Endurance Training Combined With Cognitive Remediation on Everyday Functioning, Symptoms, and Cognition in Multiepisode Schizophrenia Patients. Schizophr. Bull..

[42] Nuechterlein K.H., Ventura J., McEwen S.C., Gretchen-Doorly D., Vinogradov S., Subotnik K.L. (2016). Enhancing Cognitive Training Through Aerobic Exercise After a First Schizophrenia Episode: Theoretical Conception and Pilot Study. Schizophr. Bull..

[43] Choi J., Taylor B., Fiszdon J.M., Kurtz M.M., Tek C., Dewberry M.J., Haber L.C., Shagan D., Assaf M., Pearlson G.D. (2019). The synergistic benefits of physical and cognitive exercise in schizophrenia: Promoting motivation to enhance community effectiveness. Schizophr. Res. Cogn..

[44] Nuechterlein K.H., McEwen S.C., Ventura J., Subotnik K.L., Turner L.R., Boucher M., Casaus L.R., Distler M.G., Hayata J.N. (2022). Aerobic exercise enhances cognitive training effects in first-episode schizophrenia: Randomized clinical trial demonstrates cognitive and functional gains. Psychol. Med..

[45] Dai Y., Ding H., Lu X., Wu X., Xu C., Jiang T., Ming L., Xia Z., Song C., Shen H. (2022). CCRT and aerobic exercise: A randomised controlled study of processing speed, cognitive flexibility, and serum BDNF expression in schizophrenia. Schizophrenia.

[46] Vita A., Barlati S. (2019). The Implementation of Evidence-Based Psychiatric Rehabilitation: Challenges and Opportunities for Mental Health Services. Front. Psychiatry.

[47] Wykes T. (2018). Cognitive remediation—Where are we now and what should we do next?. Off. J. Ital. Soc. Psychopathol..

[48] Deste G., Barlati S., Cacciani P., DePeri L., Poli R., Sacchetti E., Vita A. (2015). Persistence of effectiveness of cognitive remediation interventions in schizophrenia: A 1-year follow-up study. Schizophr. Res..

[49] Horan W.P., Dolinsky M., Lee J., Kern R.S., Hellemann G., Sugar C.A., Glynn S.M., Green M.F. (2017). Social Cognitive Skills Training for Psychosis With Community-Based Training Exercises: A Randomized Controlled Trial. Schizophr. Bull..

[50] Tan S., Zhu X., Fan H., Tan Y., Yang F., Wang Z., Zhao Y., Fan F., Guo J., Li Z. (2019). Who will benefit from computerized cognitive remediation therapy? Evidence from a multisite randomized controlled study in schizophrenia. Psychol. Med..

[51] Vaskinn A., Løvgren A., Egeland M.K., Feyer F.K., Østefjells T., Andreassen O.A., Melle I., Sundet K. (2019). A randomized controlled trial of training of affect recognition (TAR) in schizophrenia shows lasting effects for theory of mind. Eur. Arch. Psychiatry Clin. Neurosci..

[52] Correll C.U., Ismail Z., McIntyre R.S., Rafeyan R., Thase M.E. (2022). Patient Functioning, Life Engagement, and Treatment Goals in Schizophrenia. J. Clin. Psychiatry.

[53] Mohr P., Galderisi S., Boyer P., Wasserman D., Arteel P., Ieven A., Karkkainen H., Pereira E., Guldemond N., Winkler P. (2018). Value of schizophrenia treatment I: The patient journey. Eur. Psychiatry.

